# A Non-severe Coronavirus Disease 2019 Patient With Persistently High Interleukin-6 Level

**DOI:** 10.3389/fpubh.2020.584552

**Published:** 2020-11-16

**Authors:** Nurfarah Lydia Hambali, Malehah Mohd Noh, Shahleni Paramasivam, Tock Hing Chua, Firdaus Hayati, Alvin Oliver Payus, Tze Yuan Tee, Khairul Taufiq Rosli, Mohammad Faruq Abd Rachman Isnadi, Benny O. Manin

**Affiliations:** ^1^Department of Pathobiology and Medical Diagnostic, Faculty of Medicine and Health Sciences, Universiti Malaysia Sabah, Kota Kinabalu, Malaysia; ^2^Medical Based Department, Faculty of Medicine and Health Sciences, Universiti Malaysia Sabah, Kota Kinabalu, Malaysia; ^3^Surgical Based Department, Faculty of Medicine and Health Sciences, Universiti Malaysia Sabah, Kota Kinabalu, Malaysia; ^4^Medical Department, Tawau Hospital, Tawau, Malaysia

**Keywords:** COVID-19, cytokine release syndrome, IL-6, HBV infection, HCC

## Abstract

Interleukin 6 (IL-6) is one of the markers of immune system activation indicating existent infection and inflammation. We present here a case of a 55-year-old male COVID-19 patient with an unusual high level of interleukin 6 (IL-6). Further investigation revealed he had hepatocellular carcinoma (HCC) with underlying hepatitis B. He did not present with respiratory symptoms although a baseline chest x-ray showed changes, and the patient was categorized as Class 3A of COVID-19. Routine investigations proceeded with high-resolution computed tomography and IL-6 to monitor for progression to severe COVID-19. Notably, there was a high IL-6 level but other parameters did not show he was in severe COVID-19. In this report, we conclude that elevated IL-6 level in a COVID-19 patient is not necessarily associated with severe COVID-19.

## Introduction

Interleukin 6 (IL-6) is one of the essential members of the cytokine network and executes a crucial part in cytokine release syndrome in coronavirus disease 2019 (COVID-19) cases, a condition where the severe acute respiratory syndrome coronavirus 2 (SARS-CoV-2) activating the immune system causing massive cytokine releases (1). Several studies have also shown that high IL-6 levels in hepatitis B-infected patients were involved in developing a hepatitis B virus (HBV)-induced liver cirrhosis and exacerbated liver injury (2). We present here a 55-year-old male COVID-19 patient with an underlying hepatitis B infection showing an unusually high level of IL-6.

## Case Description

A male oil-palm estate worker, aged 55 years, admitted after a mass COVID-19 screening was conducted in his workplace. COVID-19 was diagnosed as SARS-CoV-2 was detected in his first nasopharyngeal swab specimen by real-time reverse transcription-polymerase chain reaction assay. Subsequent history taking revealed he had abdominal distension and bilateral leg swelling for 10 days. He denied having fever, cough, upper respiratory tract infection, and loose stools. During admission, he had a temperature of 36.8°C, with a pulse of 89 beats/min, a respiratory rate of 20 breaths/min, and a blood pressure of 147/89 mmHg. His lungs were clear to auscultation, and no murmur was appreciated. Clinically, he had bilateral pedal edema until his shin, ascitic abdomen with a positive fluid thrill, unable to appreciate his liver. He was not jaundiced, and there were no other signs of chronic liver disease. He did not have any known medical history apart from being an active smoker for more than 20 years. He denied alcohol consumption and parenteral exposure (transfusion, tattoos, and injection of drug use).

His abnormal laboratory results during admission are shown in Table 1: total white cell count 9.1 10^3^/μl, his absolute neutrophil count 6.1 10^3^/μl, absolute lymphocyte count 1.5 10^3^/μl excluding lymphopenia, prothrombin time 14.2 s, activated partial thromboplastin time 36.3 s, international normalized ratio 1.4%, elevated lactate dehydrogenase 768 U/L, high level of C-reactive protein 49.9 mg/L, D-dimer level 8,570 ng/ml, and ferritin ranging from 10,599.25 μg/l. A derangement in his liver enzymes was noted: aspartate aminotransferase 125 U/L, alkaline phosphatase 210 U/L, high bilirubin level 37.1 μmol/L, and low level of albumin 23 g/L and alpha-fetoprotein (AFP) > 1,660 U/ml.

**Table 1:** Serial blood investigations of the patient.

**Variables**	**Reference range**	**Day 1 (18/4)**	**Day 3 (20/04)**	**Day 5 (22/04)**	**Day 13 (30/04)**	**Day 20 (07/05)**	**Day 23 (10/05)**	**Day 26 (13/05)**
TWC^a^	4.0–10.0 10^3^/μl	9.1	9.7	9.1	12.4	10.3	11.2	9.0
ANC^b^	2.0–7.0 10^3^/μl	6.1	6.8	6.4	10.3	8.2	9.0	6.7
ALC^c^	1.0–3.0 10^3^/μl	1.5	1.6	1.3	1.4	1.0	1.1	1.4
Albumin	35–54 g/L	23	23	21	19	21	21	23
Bilirubin	3.4–20.5 μmol/L	37.1	44.2	42.4	32.8	53.2	76.8	76.6
ALP^d^	40–150 U/L	210	198	184	186	173	195	220
ALT^e^	<55 U/L	35	36	37	58	50	50	67
AST^f^	5–34 U/L	125	124	125	152	106	136	196
PT^g^	9.0–11.3 s	14.2				15.3	16.9	
aPTT^h^	30.3–40.2 s	36.3				31.3	33.6	
INR^i^	0.8–1.4%	1.4				1.51	1.66	
LDH^j^	125–220 U/L	768	649	527	596	431		
CRP^k^	<5 mg/L	49.9	64.2	66.4	53	190.4	131.0	61.8
D-dimer	<500 ng/ml			8570				
Alpha fetoprotein	0.74–7.30 U/ml		>1,660					
Ferritin	4.63–274.66 μg/L					10599.25	11275.23	13864.00

*There was a derangement in his liver enzymes as exhibited by elevated aspartate aminotransferase and alkaline phosphatase with high bilirubin and low albumin. Coagulation studies (prothrombin time and international normalized ratio) were slightly prolonged. The inflammatory markers (lactate dehydrogenase, C-reactive protein, D-dimer, and ferritin) with high alpha-fetoprotein were also elevated. However, there was no lymphopenia (low absolute lymphocyte count) noted in this patient*.

a *Total white cell*,

b *Absolute neutrophil count*,

c *Absolute lymphocyte count*,

d *Alkaline phosphatase*,

e *Alanine transferase*,

f *Aspartate aminotransferase*,

g *Prothrombin time*,

h *Activated partial thromboplastin time*,

i *International normalized ratio*,

j *Lactate dehydrogenase*,

k *C-reactive protein*.

**Figure 1:**
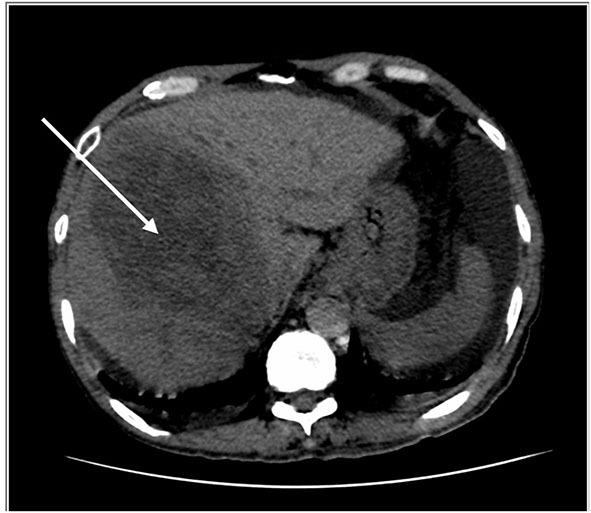
HRCT thorax, including the liver. Key images of two scans of the same patient, at the same slice taken 3 weeks apart. The initial scan shows a large right liver lobe mass (white arrow). Please note that no obvious lesion is seen in the left liver lobe.

He had no respiratory symptoms, was never on any oxygen support, and had been afebrile throughout his stay. However, his baseline chest X-ray showed changes, and the patient was categorized as class 3A. As a routine, they proceeded with high-resolution computed tomography (HRCT) thorax and IL-6 to monitor for progression for severe COVID-19. HRCT thorax (Figure 1) showed evidence of chronic lung changes with pleural effusion and centrilobular nodules, which is atypical for COVID-19. Notably, the presence of multifocal liver lesions with portal vein thrombosis. His anti-HCV test was non-reactive; however, his hepatitis B surface antigen came back as reactive. What is intriguing about this case was that, although his IL-6 level was high, other medical parameters did not show he was in severe COVID-19. We analyzed his IL-6 level in our research laboratory in Universiti Malaysia Sabah, and the result is shown in Table 2.

**Table 2:** Serial interleukin 6 (IL-6) level of the patient.

**Date**	**IL-6 level value (pg/ml) (*n* = 3)**
24/04/2020	122.0
27/04/2020	88.63
29/04/2020	37.2
03/05/2020	169
04/05/2020	144

*The IL-6 level of the patient was analyzed using Human IL-6 Quantikine Elisa Kit and run with Thermo Scientific Multiskan GO Microplate Spectrophotometer, analyzed in five subsequent dates. There is a decreasing trend for a duration of 6 days before an upsurge on day 23 (03/05/2020) of illness, much higher than the first reading, and subsequently, it fell back within a 1-day period*.

**Figure 2:**
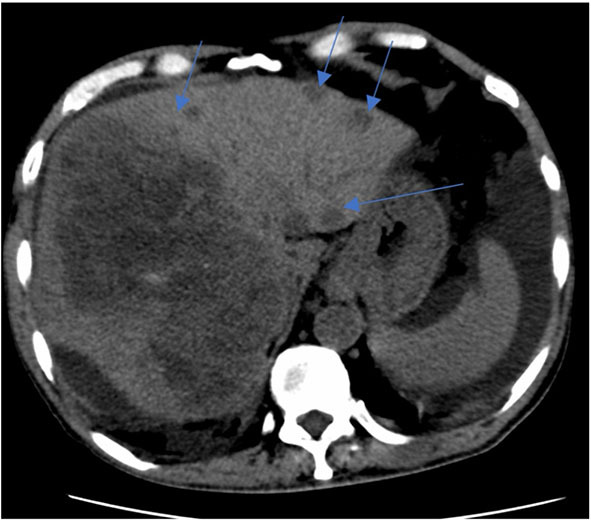
CT liver 4 phase. Key images of two scans of the same patient, at the same slice taken 3 weeks apart. Subsequent CT shows enlarging right liver mass with multiple new left liver lobe lesions (multiple blue arrows).

Peritoneal tapping was performed for diagnostic purposes, and the peritoneal fluid analysis revealed the following results: serum albumin 22, peritoneal albumin 6, and serum ascites albumin gradient 1.6 g/dl and indicates portal hypertension, gravitating toward the non-peritoneal cause of ascites. With a reactive hepatitis B and findings from HRCT showing liver lesions, they ensue four-phase liver computed tomography. A comparison with the previous scan (shown in Figures 1, 2) showed multiple enlarging and new liver lesions with no signs of tumoral rupture. The liver margin is irregular, more pronounced in the left liver lobe, indicating liver cirrhosis. With the underlying hepatitis B liver cirrhosis and raised AFP, the likely lesions represent hepatocellular carcinoma (HCC) or metastases in disease progression.

He was given tab spironolactone 37.5 mg once a day (OD) for his ascites due to cirrhosis, tab tenofovir 300 mg OD for his hepatitis B infection, tab pantoprazole 40 mg OD, and tab zincofer 1 tab OD for his anemia. He was also on fluid restriction of 1 L/day. However, liver biopsy was not done because of his unregistered visitor status and financial constraint; the hepatobiliary and gastroenterology team decided for conservative management. He did not have a fever or develop any oxygen desaturation during his hospital stay. He was tested negative for COVID-19 in subsequent tests on days 20, 25, and 32 of illness. Before discharge, clinically, he was well, did not have respiratory symptoms, and the vital signs were normal. He was supplied with medications until the next follow-up visit 2 months later.

## Discussion of Case Study

Clinical presentations of COVID-19 varied from mild flu-like symptoms to life-threatening respiratory distress and multiorgan dysfunction. Some individuals will experience cytokine release syndrome (CRS), a hyper-inflammation caused by immense cytokines production by an overly activated immune system. IL-6 plays an essential role in mediating CRS-induced damage, and higher IL-6 levels were correlated with the severity of the disease.

Liu et al. (3) have proposed utilizing the IL-6 blockade to manage COVID-19-induced CRS. However, before it can be used in clinical application, much more research needs to be done to set the following criteria: diagnosis criteria of CRS, disease severity grading system, combined blockade with antiviral treatment, secondary infection monitoring system, and cytokine measurement and using IL-6 levels as biomarkers. Nevertheless, there are also valid concerns that such therapy may have potential harm to the patients (4). This is because IL-6 has significant anti-inflammatory properties and may play a beneficial role for IL-6 in the host response to infection. Clinical data on using IL-6 receptor antagonists such as tocilizumab indicated an increase in severe and opportunistic infections. Thus, until more information is available, caution should be exercised in prescribing IL-6 blockade to COVID-19 patients.

The patient denied he had any respiratory symptoms and had been stable under room air. HRCT and IL-6 were done after an abnormal chest X-ray. In view of his high IL-6 level, we had to determine whether he was in a severe COVID-19 phase. However, his clinical status, other laboratory parameters, and imaging had ruled against a cytokine storm or severe COVID-19 pneumonia. This patient was categorized as phase 3A due to his lung findings, although the lung findings were atypical of COVID-19 pneumonia. The majority of COVID-19 patients presented with lung involvement, as exhibited by chest radiography, and show either unilateral or bilateral infiltrate or consolidation (5, 6).

Furthermore, his IL-6 levels indicated a fluctuation within a period of 11 days (Table 2). There was a decreasing trend for a duration of 6 days before an upsurge on day 23 of illness, much higher than the first reading, and subsequently, it fell back within a 1-day period, which does not correlate with his COVID-19 disease progression. His third nasopharyngeal swab on day 20 of illness also tested negative for COVID-19. Reviewing his other laboratory parameters, he did not exhibit any lymphopenia or thrombocytopenia, which is typical of COVID-19 illness. Lymphopenia can be due to the suppression of normal T cell activation by the presence of IL-6 (7). Thus, we conclude the patient was not in a severe COVID-19 phase. However, his C-reactive protein, D-dimer, lactate dehydrogenase, and AFP were elevated with a derangement in his liver enzymes and coagulation profile. His underlying Hep B infection could cause this. Ascites fluid also increased fibrinolytic activity, which might have elevated his D-dimer level.

Several previous studies by Xia et al. (8) evaluate how the HBV infection could upregulate the IL-6 level and further aggravate the underlying liver pathologies to the progression of HCC. This could be elucidated by the presence of the HBV X protein (HBx). IL-6 synthesis has a strict transcriptional and posttranscriptional regulation affected by many factors. For example, the HBx gene increases nuclear factor kappa B (NF-κB) and/or NF-IL6 DNA binding activity, and HBx transfection also can induce IL-6 expressions (8). Both can subsequently increase IL-6 levels in an HBV-infected liver microenvironment.

When we did a comparison of the IL-6 concentration between this case with the other nine COVID-19 monoinfected patients, the levels in the latter were much lower. IL-6 levels of the COVID-19 monoinfected patients were ranged from <3.13 to 15.8 ng/ml (*n* = 3). The lowest reading of this COVID-19/HBV coinfected was 37.2 ng/ml, with the highest being 169 ng/ml (*n* = 3).

Furthermore, IL-6 levels increase with HBx expressions in hepatocytes and hepatoma cells, which occurs in a MyD88-dependent manner as shown by Xia et al. (8). They introduced a chemical agent named diethylnitrosamine into the mice, causing hepatocyte necrosis resulting in the production of various macromolecules, which then stimulate the Toll-like receptors (TLRs) activating macrophages. TLRs then activate MyD88, followed by NF-κB translocation to the nucleus and IL-6 induction. Hence, the TLR–Myd88–NF-κB–IL-6 signaling pathway has a crucial part in liver tumor generation and leads to hepatitis B progression to cirrhosis or HCC.

For our case, his clinical manifestation and imaging features, the derangement in his liver enzymes, and elevation of his AFP with reactive Hep B infection indicate the possibility of the progression to HCC. A combination of elevation of IL-6 and AFP has moderate accuracy in predicting the future HCC development in chronic hepatitis B patients (9). The limitation of this study was that liver biopsy for the confirmatory diagnosis of HCC was not done, as he had an unregistered status and financial constraint.

## Conclusion

In conclusion, this case report demonstrates that although high serum IL-6 level reflects an ongoing inflammatory process and severe spectrum of presentation in symptomatic COVID-19 patients, it can also cause by another condition such as hepatitis or HCC, which can co-present in a COVID-19 patient. Therefore, this makes the reliability of a serum IL-6 level for diagnosing and monitoring in such patients doubtful. Our patient with a history of underlying hepatitis B infection with ascites, elevated AFP and liver function test led to an incidental finding of liver pathology, which is most likely HCC or metastatic lesions. This condition has eventually resulted in the elevation of his IL-6 level, further aggravating his inflammation process. We have excluded severe COVID-19 pneumonia, as he did not have a fever and respiratory symptoms, subsequent repeated tests were negative for COVID-19, but had other parameters that could be explained by his liver pathologies. Additionally, with his preexisting liver disease and systemic immunocompromised status, he was susceptible to infections such as COVID-19.

## Data Availability Statement

All datasets generated for this study are included in the article/supplementary material.

## Ethics Statement

The studies involving human participants were reviewed and approved by Medical Review & Ethics Committee (MREC) (NMRR-20-729-54610) of Ministry of Health Malaysia. The patients/participants provided their written informed consent to participate in this study. Written informed consent was obtained from the individual(s) for the publication of any potentially identifiable images or data included in this article.

## Author Contributions

NH: full writing of the publication, assist in the sampling analysis and processing. MM: identifying all necessary people involved, communicating with physicians on case details, major editing and sampling process, analysis, and certifying results. SP: major editing and sampling process. TC: major editing and additional writing, analyzing the IL-6 samples, and certifying the result. FH and AP: editing process. TT and KR: managing physician and consultant, provided necessary details. MA and BM: analyzing and processing samples of IL-6. All authors contributed to the article and approved the submitted version.

## Conflict of Interest

The authors declare that the research was conducted in the absence of any commercial or financial relationships that could be construed as a potential conflict of interest.
